# Blood Eosinophil Subtype-Specific Protein Gene Expression and Eosinophil-Derived Serum Mediators in Allergic Asthma Patients After Bronchial Challenge with *Dermatophagoides pteronyssinus*

**DOI:** 10.3390/cells15171579

**Published:** 2026-08-30

**Authors:** Airidas Rimkunas, Andrius Januskevicius, Egle Vasyle, Jolita Palacionyte, Skaidrius Miliauskas, Kestutis Malakauskas

**Affiliations:** 1 Laboratory of Pulmonology, Department of Pulmonology, Lithuanian University of Health Sciences, LT-44307 Kaunas, Lithuania; andrius.januskevicius@lsmu.lt (A.J.); egle.vasyle@lsmu.lt (E.V.); kestutis.malakauskas@lsmu.lt (K.M.); 2 Department of Pulmonology, Lithuanian University of Health Sciences, LT-44307 Kaunas, Lithuania; jolita.palacionyte@lsmu.lt (J.P.); skaidrius.miliauskas@lsmu.lt (S.M.)

**Keywords:** allergic asthma, eosinophil subtypes, bronchial allergen challenge, eosinophilic inflammation, eosinophil gene expression, eosinophil-derived mediators

## Abstract

Background: Differences in gene expression between inflammatory-like (iEOS-like) and resident-like (rEOS-like) eosinophil subtypes, and in eosinophil-derived serum mediators, may reflect eosinophil functional activity and their potential role in the pathogenesis of allergic asthma (AA). Methods: Twenty-three patients with non-severe AA and thirteen healthy subjects (HS) were examined. AA patients underwent a bronchial allergen challenge (BAC) with *Dermatophagoides pteronyssinus* and were re-evaluated 24 h later. Blood eosinophils were isolated by gradient centrifugation and magnetic separation, followed by subtyping based on CD62L expression. Gene expression was assessed by TaqMan-based quantitative PCR. Serum eosinophil cationic protein (ECP), eosinophil-derived neurotoxin (EDN), Galectin-10 (Gal10), and NADPH oxidase 2 (NOX2) were measured using ELISA. Results: Blood eosinophil subtypes from AA patients showed significantly higher expression of *CLC*, *ECP*, *EPX*, *EDN*, *MBP*, *ALOX5*, *NOX2*, and *TGF-β1* compared to those from HS (*p* < 0.05), with no significant changes in *LTA4H* and *LTC4S*. iEOS-like cells in AA patients exhibited higher *CLC* and *EPX* expression compared to rEOS-like cells, (*p* < 0.05). Following BAC, *CLC*, *MBP*, and *TGF-β1* expression increased in both eosinophil subtypes, while *EPX* and *NOX2* increased only in iEOS-like cells (all *p* < 0.05). Serum ECP, EDN, and Gal10 concentrations were elevated in AA compared to HS and further increased after BAC (all *p* < 0.05); serum NOX2 remained unchanged. Conclusions: BAC induces a late-phase eosinophilic response in AA characterized by a partially eosinophil subtype-specific increase in gene expression and in circulating eosinophil-derived mediators. An increase in Gal10, observed at the *CLC* transcript level in eosinophils and in serum—but not in ECP or EDN—suggests the existence of mediator-specific mechanisms that regulate gene expression and extracellular release.

## 1. Introduction

Asthma comprises diverse inflammatory endotypes characterized by variable airway obstruction, airway hyper-responsiveness (AHR), and airway remodeling [1,2]. Disease symptoms can worsen periodically when a person with asthma is exposed to allergens, respiratory infections, pollutants, occupational irritants, or certain medications [3]. In allergic asthma (AA), exposure to aeroallergens activates airway epithelial and dendritic cell responses that drive type 2 inflammation. Dendritic cells process allergen-derived antigens and present them to naive T cells [4]. Allergen-derived proteases may stimulate epithelial release of the alarmins thymic stromal lymphopoietin (TSLP), interleukin (IL)-25, and IL-33, thereby promoting eosinophilic airway inflammation [5,6]. Persistent type 2 inflammation predominates in asthma and involves type 2 ILCs and type 2 T helper (Th2) cells, which produce IL-4, IL-5, and IL-13, thereby further contributing to the inflammatory cascade. IL-4 and IL-13 promote B-cell class switching and immunoglobulin (Ig)E production, barrier disruption, goblet-cell hyperplasia, mucus production, and local tissue remodeling, while IL-5 drives eosinophil progenitor differentiation and maturation in bone marrow, inflammatory cell trafficking, and survival [7,8].

Activated eosinophils may undergo transcriptional and phenotypic changes that enhance their trafficking and mediator release [9]. Cytokines produced by injured epithelial cells and local immune cells can further stimulate eosinophils and promote degranulation, the release of pro-inflammatory mediators, including eosinophil-derived neurotoxin (EDN, *RNASE2*), eosinophil cationic protein (ECP, *RNASE3*), major basic protein (MBP, *PRG2*), eosinophil peroxidase (EPX), enzymes, chemokines, and growth factors such as transforming growth factor beta 1 (TGF-β1) [9,10]. In addition to the well-established granule-derived proteins, eosinophils may store and secrete cysteinyl leukotrienes, potent lipid mediators involved in mucus production, vascular permeability, and bronchoconstriction [10,11,12]. Therefore, peripheral blood eosinophils may actively contribute to ongoing airway inflammation via eosinophil-derived mediators, which can cause local tissue damage, mast cell degranulation, fibroblast proliferation, and production of extracellular matrix components, leading to airway eosinophilia, increased mucus production, and airway remodeling [8,13,14,15].

Growing evidence suggests that eosinophils comprise phenotypically and functionally heterogeneous cell populations with varying activation states rather than a single terminal effector cell type. Mesnil and colleagues characterized eosinophil subpopulations using immunophenotyping in a mouse model, identifying and describing resident eosinophils (rEOS) as a distinct cell subpopulation compared with inflammatory eosinophils (iEOS) [16]. Subsequent studies in humans have suggested that circulating eosinophil populations may exhibit heterogeneous activation states [17] associated with disease severity [18], response to biologic therapy [19,20,21], and distribution across distinct airway diseases [22,23], highlighting the need for further eosinophil subtype-based studies. Despite increasing recognition of eosinophil heterogeneity in asthma, data remain limited on how allergen-induced inflammatory responses influence eosinophil biology during disease exacerbations and progression.

The purpose of this study was to investigate allergen-induced changes in the expression of granule protein genes in blood eosinophil subtypes and in concentrations of circulating eosinophil-derived mediators in allergic asthma (AA).

## 2. Materials and Methods

### 2.1. Study Design, Population, and Experimental Plan

A total of 36 adult subjects were included in this study. Participants were adult men and women aged 18–66. All study participants were patients in the Department of Pulmonology at the Lithuanian University of Health Sciences (LUHS) Hospital, Kauno Klinikos, and were recruited between December 2023 and December 2025. The study followed the Declaration of Helsinki and received approval from the Kaunas Regional Biomedical Research Ethics Committee of the Lithuanian University of Health Sciences (protocol code P3-BE-2-58/2020, approved 28 September 2023). Each participant underwent a comprehensive health history and physical examination, a standardized allergy skin prick test, and spirometry to confirm eligibility. After the initial examination, those who met the study’s inclusion criteria were informed of the study requirements, introduced to the study protocol, and asked to sign an informed written consent form. The AA group also underwent a methacholine challenge test. Likewise, we recruited healthy control subjects (HS) at Hospital Kauno Klinikos through a hospital-based biomedical study recruitment campaign. Clinicians screened responders during their initial visit. If deemed eligible, non-atopic individuals who met the criteria described in Table 1 were invited to participate.

After admission, we conducted a follow-up experimental visit within 4 weeks. Participants first underwent fractional exhaled nitric oxide (FeNO) testing, then lung spirometry, and blood samples were drawn for complete blood count (CBC), total serum IgE, and isolation of blood eosinophil subtypes for experimentation. In the AA group, we performed a bronchial allergen challenge (BAC) with *Dermatophagoides pteronyssinus* (*D. pteronyssinus*) and repeated all clinical and experimental tests 24 h later during a new experimental visit.

Following isolation of blood eosinophils, samples with cell purity < 96% and viability < 94% were excluded from the study. All data analyzed and presented in the manuscript were from individual samples that passed these criteria.

### 2.2. Complete Blood Count and Serum Total Immunoglobulin E

Investigated subjects’ blood samples were collected in sterile vacutainers (BD Vacutainer Systems, Plymouth, UK) and transported directly to the LUHS Hospital Kauno Klinikos laboratory. CBC analysis, including leukocyte differentiation, was performed by hematology analyzer UniCel^®^ DxH 800 Coulter^®^ Cellular Analysis System (Beckman Coulter, Miami, FL, USA). Serum samples were prepared according to standard diagnostic laboratory procedures, and total serum IgE concentrations were measured by immunoassay analyzer AIA-2000 (Tosoh Bioscience, South San Francisco, CA, USA).

### 2.3. Fractional Exhaled Nitric Oxide Measurement

FeNO was measured using a handheld Vivatmo device (Bosch Healthcare Solutions, Waiblingen, Germany) according to the manufacturer’s instructions. All measurements were performed in the hospital during the experimental visits, under supervision of trained clinical personnel. The device was sanitized, and a brief self-calibration was performed between uses. Participants were instructed to blow steadily through the single-use mouthpiece for about 5 s. At least two acceptable measurements were obtained, single-use equipment was properly discarded, and the mean FeNO value was calculated.

### 2.4. Allergy Skin Prick Test

The allergen skin prick test was done during each study subject’s initial screening visit to confirm inclusion into the study criteria. Each individual’s forearm was exposed to house dust mite (HDM), *Dermatophagoides pteronyssinus* (*D. pteronyssinus*) and other clinically relevant aeroallergens using standardized skin test allergen extracts (Stallergenes, S.A., Antony, France) and sterile, single-use lancets. In addition, a negative control (saline) and a positive control (1% histamine dihydrochloride solution) were included in each test. Skin prick test results were interpreted 15 min after application. Sensitization to the respective allergens was considered positive if the measured mean wheal diameter was ≥3 mm greater than that of the negative control.

### 2.5. Lung Function and Airway Hyperresponsiveness Testing

Each investigated subject underwent spirometry testing using an ultrasonic spirometer (Ganshorn Medizin Electronic, Niederlauer, Germany). The procedure was performed after FeNO measurements. Forced vital capacity (FVC), forced expiratory volume in 1 s (FEV_1_), and the FEV_1_/FVC ratio were measured. At least three acceptable and reproducible forced expirations were recorded and selected for analysis, as previously described [24].

The methacholine challenge test was used to evaluate AHR. Individuals comprising the AA group were asked to inhale increasing concentrations of aerosolized methacholine at two-minute intervals using a pressure dosimeter (Provo.X, Ganshorn Medizin Electronic, Niederlauer, Germany) until their FEV_1_ decreased by 20% from baseline (PD20_m_), as previously described [24].

### 2.6. Bronchial Allergen Challenge Test

The bronchial allergen challenge test was performed on all study participants in the AA group at the end of their first experimental visit using inhaled *D. pteronyssinus* allergen (DIATER, Madrid, Spain). We started assessing the bronchoconstriction effect 2 min after nebulized saline inhalation. Investigated individuals inhaled the allergen at 10 min intervals, starting with a 0.1 histamine-equivalent prick (HEP)/mL allergen concentration and increasing it to 1.0, 10.0, 20.0, 40.0, and 60.0 HEP/mL via a pressure dosimeter (Provo.X, Ganshorn Medizin Electronic, Niederlauer, Germany). The procedure would be interrupted after achieving a ≥20% reduction in patients’ FEV_1_ (L) from their baseline. Successfully provoked individuals would then return 24 h later for the second experimental visit.

### 2.7. Peripheral Blood Granulocyte Separation and Eosinophil Isolation, Purification, and Subtyping

Peripheral blood samples were collected into sterile Vacutainer tubes containing K2EDTA (BD Vacutainer Systems). Blood samples were mixed with phosphate-buffered saline (GIBCO, Paisley, UK) and carefully layered onto Ficoll-Paque Premium (Cytiva, Uppsala, Sweden) density gradient medium, then centrifuged at 400× *g* for 30 min at room temperature. Further details on granulocyte separation, eosinophil isolation, and subtyping into iEOS-like and rEOS-like cells are described in detail [24]. Following blood eosinophil subtype separation using magnetic microbeads conjugated with antibodies against surface CD62L and a magnetic separation column (Miltenyi Biotec, Bergisch Gladbach, Germany), samples from iEOS-like and rEOS-like cell fractions were analyzed by flow cytometry (FACS Calibur, BD Biosciences, San Jose, CA, USA), to verify the efficiency of magnetic separation, as previously shown and described [25]. In addition, an automated cell counter (ADAM, NanoEnTek Inc., Mountain View, CA, USA) was used to count total cell numbers and viability.

### 2.8. Blood Eosinophil Subtype RNA Isolation and Gene Expression Analysis

After separating and evaluating blood eosinophil subtypes, cells were lysed using TRIzol reagent (Invitrogen, Paisley, UK). A miRNeasy Mini Kit (Qiagen, Germantown, MD, USA) was used to purify total RNA, and an additional genomic DNA digestion step using RNase-free DNase set (Qiagen) was included, all according to the manufacturer’s instructions. A microvolume spectrophotometer, the NanoDrop 2000c (Thermo Fisher Scientific, Wilgminton, DE, USA), was used to evaluate RNA concentration and purity. A high-capacity RNA-to-cDNA kit (Applied Biosystems by Thermo Fisher Scientific, Vilnius, Lithuania) was then used to reverse-transcribe the sample RNA into complementary DNA (cDNA). For gene expression analysis, custom TaqMan^®^ Array Fast Plates (Applied Biosystems by Thermo Fisher Scientific, Vilnius, Lithuania) and quantitative reverse transcriptase polymerase chain reaction (qRT-PCR) were performed on the 7500 Fast Real-Time PCR System (Applied Biosystems, Foster City, CA, USA) according to the manufacturer’s instructions. To normalize the data, two endogenous control genes, 18S ribosomal RNA (18S rRNA) and actin beta (ACTB), were used. AA patients’ blood eosinophil subtype gene expression is presented as logarithm-transformed fold changes (2^−ΔΔCt^) relative to the HS eosinophil subtype. Similarly, in the AA group, gene expression at the 2nd visit (after BAC) was calculated relative to baseline (1st visit). Because the blood eosinophil subtype gene expression analysis required multiple purifications and separations, RNA isolation, and a sufficient yield, this analysis was performed in a representative group of study participants with adequate eosinophil recovery and sample quality.

### 2.9. Serum Mediator Concentration Evaluation

Blood serum levels of ECP, EDN (Elabscience Bionovation Inc., Houston, TX, USA), Gal10 (Antibodies.com Limited, Cambridge, UK), and NOX2 (Elk Biotechnology Co., Ltd., Sugar Land, TX, USA) were measured by the enzyme-linked immunosorbent assay (ELISA). Peripheral blood from the investigated individuals was collected into blood collection tubes (BD Vacutainer SST II Advance, Becton Dickinson, Plymouth, UK) and allowed to clot for 30 min. Afterward, the collection tubes were centrifuged at 1300× *g* for 10 min at room temperature to separate the serum from clotted blood. The prepared serum samples were immediately transferred to 2 mL cryogenic tubes (TPP, Trasadingen, Switzerland) and frozen at −80 °C for subsequent protein-level analysis. The ELISA was performed according to each manufacturer’s instructions. After incubation with diluted blood serum samples and sample standards, the plates were washed, incubated with biotinylated detection antibodies, washed again, and a streptavidin-horseradish peroxidase conjugate was added to the antibody–antigen–antibody complex. A final wash was performed, and a chromogenic substrate, 3,3′,5,5′-tetramethylbenzidine, was used to produce a blue-colored product whose intensity is proportional to the amount of analyte in the sample. A sulfuric acid-based STOP solution was added to halt the enzymatic reaction (changing the color to yellow), and the optical density (OD) was measured at 450 nm using a microplate spectrophotometer (BioTek Epoch™, Winooski, VT, USA). The measured OD values were corrected by subtracting the mean blank well OD, and sample concentrations were calculated from the standard curve for each analyte, adjusted for dilution factors, and expressed as final protein concentrations per 1 mL of blood serum.

### 2.10. Statistical Analysis

Statistical analyses were performed using GraphPad Prism 10 for Windows (GraphPad Software, Inc., San Diego, CA, USA). Results are presented as mean ± standard error of the mean (SEM), or as described within data tables. The assumption of normality in the data distribution was assessed using the Shapiro–Wilk normality test. Several variables deviated from the assumption of normality; thus, nonparametric tests were used throughout the analysis. Differences between the two independent groups were evaluated using the two-sided Mann–Whitney U test. The Wilcoxon matched-pairs signed-rank test was used to compare two dependent groups. The correlation analyses were performed using Spearman’s rank correlation test. Finally, *p* < 0.05 was considered statistically significant.

## 3. Results

### 3.1. Study Subject Characteristics

This study included 23 individuals with non-severe, steroid-free, sensitization to at least the *D. pteronyssinus* allergen, comprising allergic asthma (AA) group, and 13 HS who served as the control group. Table 2 shows the demographic and clinical characteristics of the study population. No significant differences in age, BMI, or sex distribution were observed between the AA and HS groups (*p* > 0.05).

Peripheral blood eosinophil counts, fractional exhaled nitric oxide (FeNO), and total immunoglobulin E (IgE) in AA patients were significantly higher (all *p* < 0.05) than in the HS group. In addition, asthmatic patients had significantly worse lung function (FEV_1_ in L and % pred.) than the HS group, whereas FVC (in L and % pred.) displayed a non-statistically significant trend towards lower values. All AA group patients were allergic to HDM, showed airway hyperresponsiveness during the methacholine challenge, and had elevated total serum IgE levels compared with the HS group.

AA patients’ FEV_1_ and FVC (in liters and percentage of predicted) decreased significantly, indicating physiological airway obstruction, while the blood eosinophil count and FeNO increased 24 h after BAC with house dust mite allergen (all *p* < 0.05), with no changes in total IgE. These observed changes were consistent with the successful induction of a late-phase, allergen-driven eosinophilic inflammatory response following BAC [26].

### 3.2. Blood Eosinophil Subtype Gene Expression

We investigated the gene expression of secondary eosinophil granule proteins—ECP (*RNASE3*), EDN (*RNASE2*), EPX, MBP (*PRG2*), and CLC. In addition, cysteinyl leukotrienes have been implicated as mediators of inflammation in human bronchial asthma, contributing to airway remodeling [12]; thus, the expression of the biosynthesis-related enzymes leukotriene C4 synthase (LTC4S), leukotriene A4 hydrolase (LTA4H), and arachidonate 5-lipoxygenase (ALOX5) was also investigated. Nicotinamide adenine dinucleotide phosphate oxidase 2 (NOX2) produces superoxide, a critical ROS involved in innate and adaptive immunity, and TGF-β1 is a major mediator of pro-inflammatory responses [27]; thus, their expression was also investigated.

**Figure 1:**
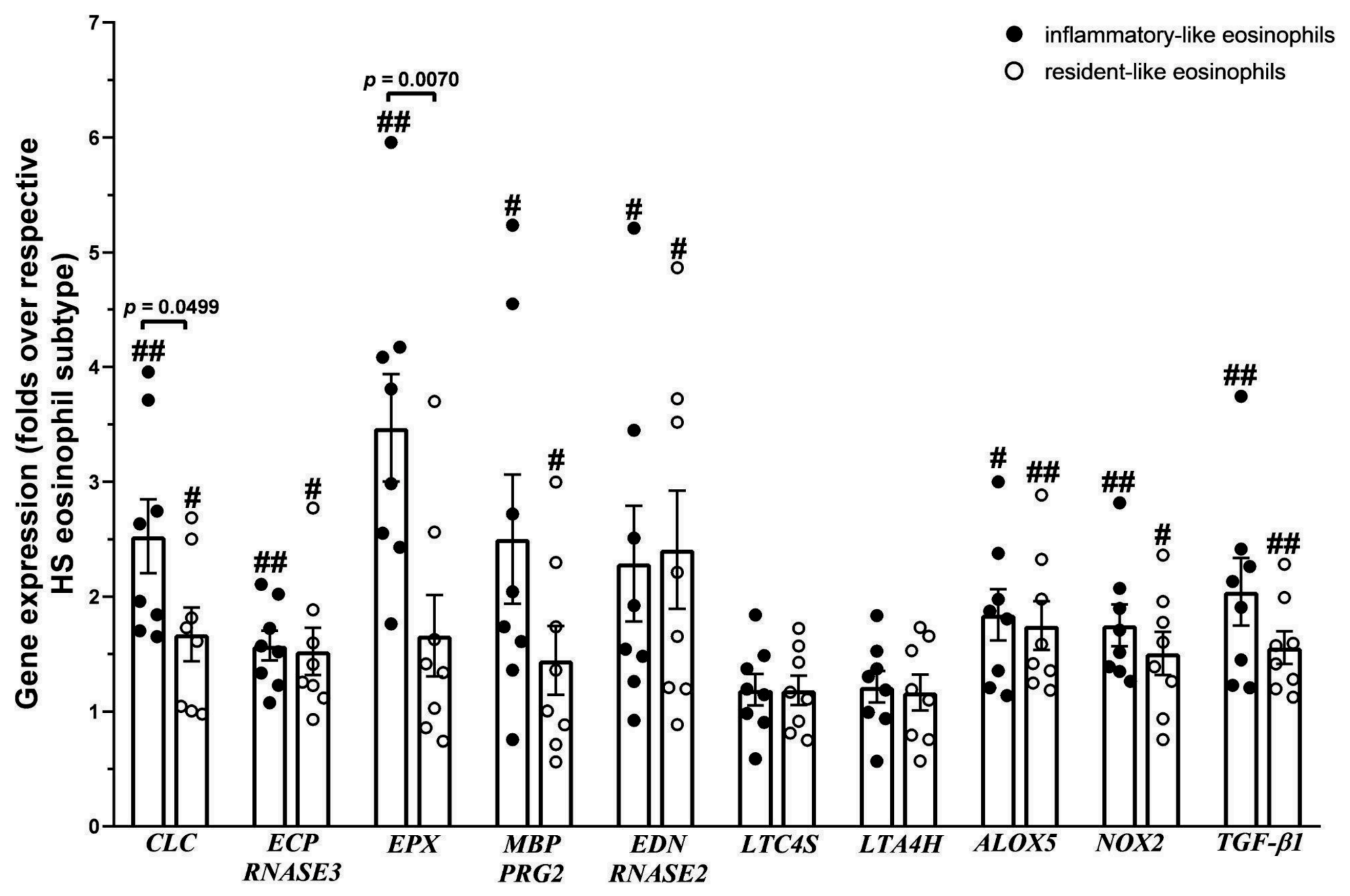
Asthmatic patients’ blood eosinophil subtype granule protein gene expression. Allergic asthma *n* = 8, healthy subjects *n* = 8. Results are shown as mean ± SEM, expressed as fold change relative to the respective healthy subject group’s blood eosinophil subtype gene expression. # *p* < 0.05. ## *p* < 0.01—compared with the respective blood eosinophil subtype of the healthy subject group. AA—allergic asthma; EPX—eosinophil peroxidase; ECP (RNASE3)—eosinophil cationic protein; EDN (RNASE2)—eosinophil-derived neurotoxin; MBP (PRG2)—major basic protein; CLC—Charcot-Leyden crystal; ALOX5—arachidonate 5-lipoxygenase; LTC4S—leukotriene C4 synthase; LTA4H—leukotriene A4 hydrolase; TGF-β1—Transforming growth factor-beta 1; NOX2—NADPH oxidase 2.

Blood iEOS-like and rEOS-like cells isolated from AA patients showed higher expression of *CLC*, *ECP*, *EPX*, *EDN*, *MBP*, *ALOX5*, *NOX2*, and *TGF-β1* (*p* < 0.05) than their respective eosinophil subtypes in the HS group (Figure 1). No significant changes in *LTC4S* and *LTA4H* expression between groups were observed.

Among eosinophil subtypes, iEOS-like cells from AA patients exhibited significantly higher *CLC* and *EPX* expression than rEOS-like cells (*p* < 0.05).

**Figure 2:**
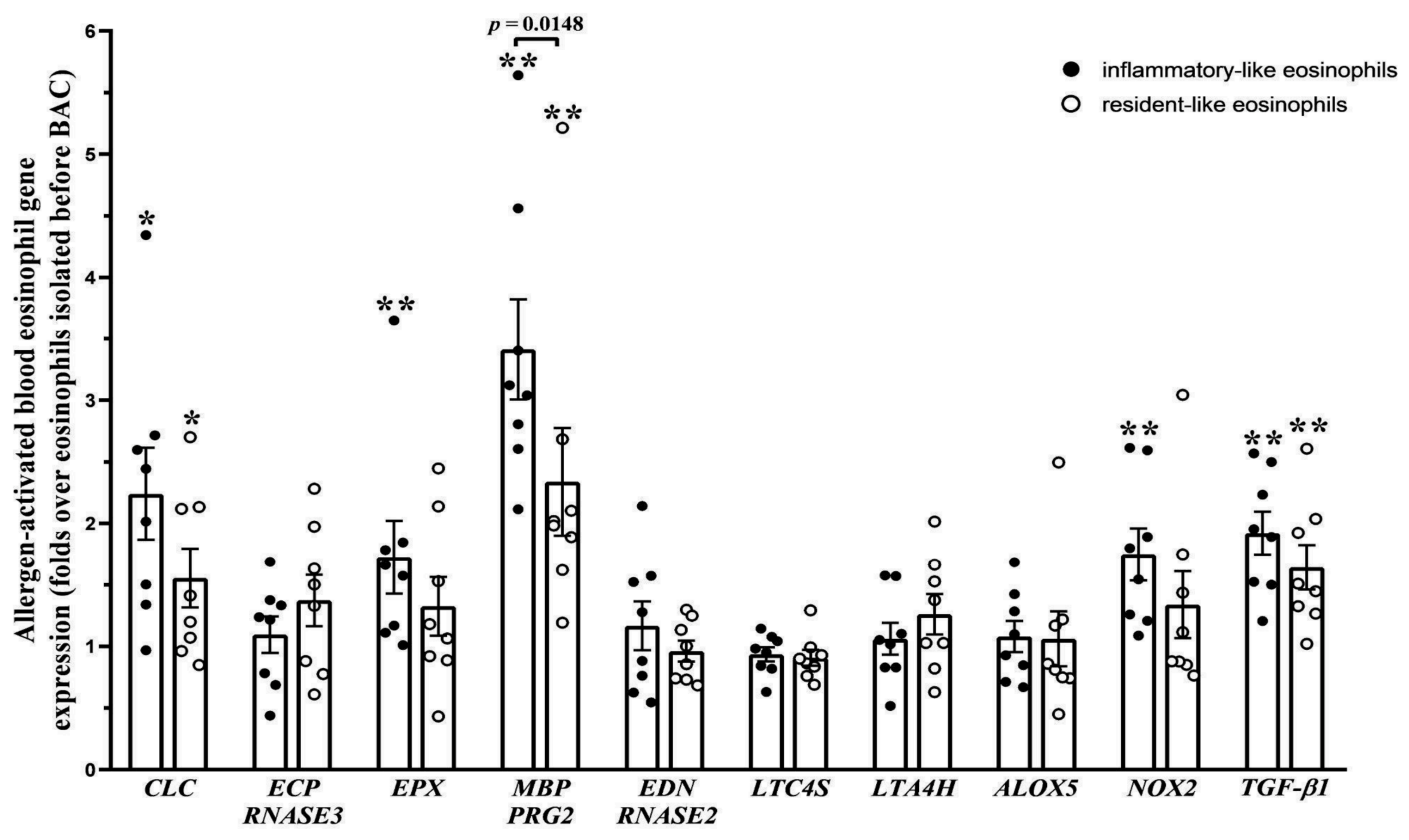
Allergic asthma patients’ blood eosinophil subtype gene expression 24 h after bronchial allergen challenge. Results are shown as mean ± SEM, with fold change relative to the non-activated blood eosinophil subtype gene expression of the investigated subjects. Allergic asthma *n* = 8. * *p* < 0.05. ** *p* < 0.01—compared with the baseline eosinophil gene expression in AA patients. AA—allergic asthma; EPX—eosinophil peroxidase; ECP (RNASE3)—eosinophil cationic protein; EDN—eosinophil-derived neurotoxin; MBP—major basic protein; CLC—Charcot-Leyden crystal; ALOX5—arachidonate 5-lipoxygenase; LTC4S—leukotriene C4 synthase; LTA4H—leukotriene A4 hydrolase; TGF-β1—Transforming growth factor-beta 1; NOX2—NADPH oxidase 2.

We also compared gene expression in blood eosinophils from AA patients before and 24 h after BAC with HDM allergen. Eosinophil subtype *CLC*, *MBP*, and *TGF-β1* gene expression in AA patients was significantly higher after BAC (*p* < 0.05), whereas *EPX* and *NOX2* expression increased significantly only in iEOS-like cells (*p* < 0.05), as shown in Figure 2. Additionally, differences between blood eosinophil subtypes were observed: iEOS-like cells showed higher *MBP* gene expression (3.4 ± 0.6 vs. 2.3 ± 0.4-fold, *p* = 0.0148) than rEOS-like cells. Similar trends were observed in *CLC* and *NOX2* expression; however, no statistically significant differences were detected.

### 3.3. Circulating Eosinophil-Derived Serum Mediators and Recovered Eosinophil Subtype Distribution Analysis

Reliable identification of circulating biomarkers remains an important task in asthma research because currently available markers incompletely capture disease activity and inflammation [28]. In this study, EDN, ECP, and Gal10 were selected for investigation based on their involvement in key pathogenic mechanisms, including eosinophil activation, degranulation, and CLC formation [10,29,30,31]. Meanwhile, growing evidence suggests that NOX2-mediated ROS production may contribute to inflammation in asthma [32,33], but its utility as a circulating biomarker is insufficiently characterized; therefore, it was also included in this study. Circulating eosinophil subtypes exhibit distinct activation states and biological properties [17,34], prompting us to investigate whether these serum mediator concentrations could reflect exacerbations or be associated with the distribution of blood iEOS-like and rEOS-like cells in asthma.

**Figure 3:**
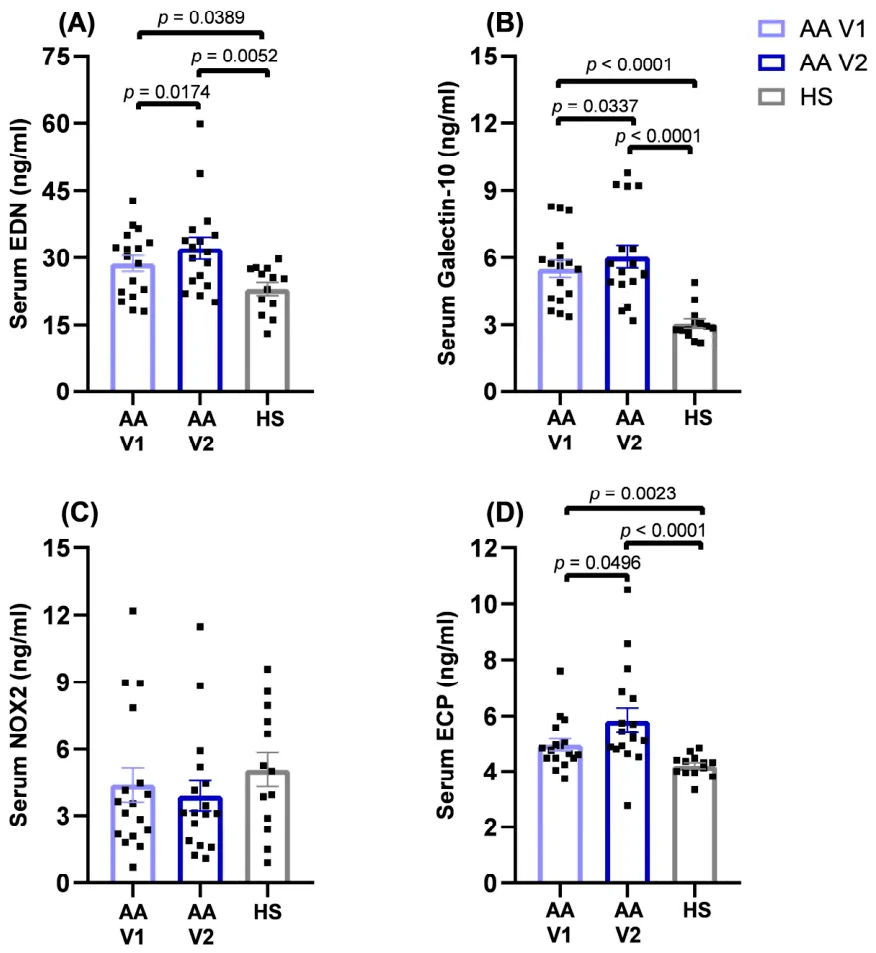
Serum mediator concentrations. (**A**) Eosinophil-derived neurotoxin (EDN). (**B**) Galectin-10 (Gal10). (**C**) Nicotinamide adenine dinucleotide phosphate oxidase 2 (NOX2). (**D**) Eosinophil cationic protein (ECP). Allergic asthma (AA) *n* = 17. Healthy subject (HS) *n* = 13. V1—allergic asthma patient’s first experimental visit. V2—allergic asthma patient’s 2nd experimental visit, 24 h after a bronchial allergen challenge with house dust mite.

Following the blood serum ELISA analysis, it was found that at baseline, serum ECP, EDN, and Gal10 concentrations in AA patients were significantly higher than in the HS group (all *p* < 0.05). No significant differences in serum NOX2 concentration were observed between groups. At the AA patient follow-up visit 24 h after BAC (V2), serum ECP, EDN, and CLC concentrations increased further (all *p* < 0.05) compared with V1 (baseline) (Figure 3). Additional data regarding serum mediator concentration analysis are provided in Appendix A.

**Figure 4:**
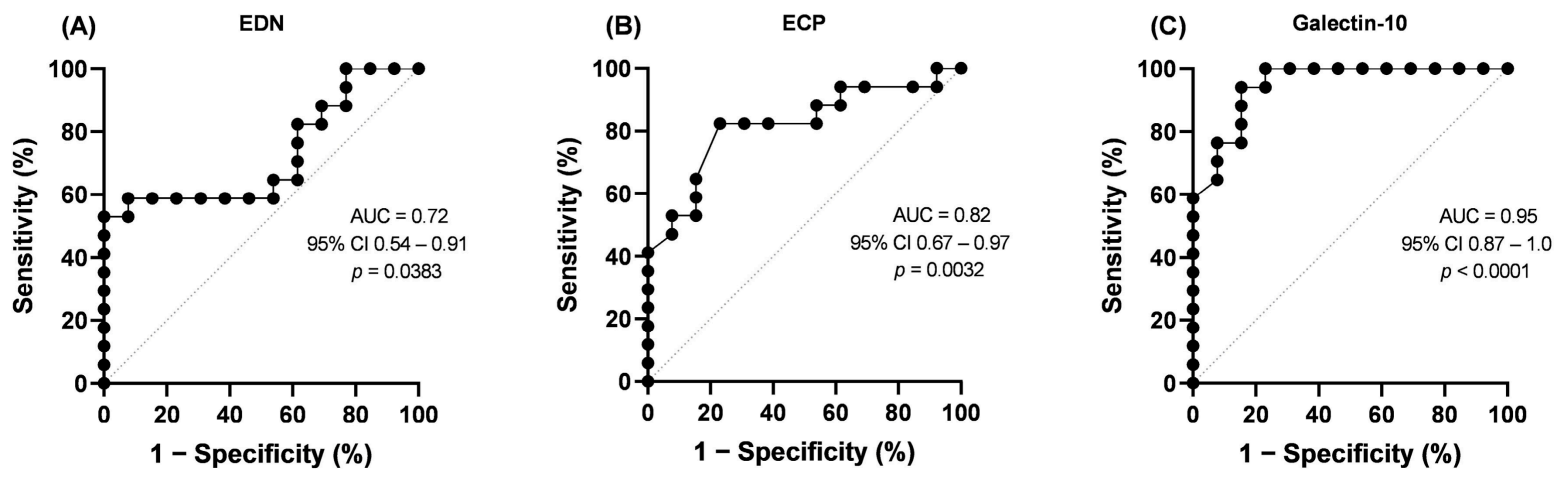
Receiver operating characteristic curve analysis of AA patients’ serum concentrations at baseline compared with those of healthy subjects. (**A**) Eosinophil-derived neurotoxin (EDN) analysis. (**B**) Eosinophil cationic protein (ECP) analysis. (**C**) Galectin-10 analysis. Allergic asthma *n* = 17, healthy subjects *n* = 13. AUC—area under the curve. CI—confidence interval.

In addition, to investigate the potential discriminatory performance of serum mediators that differed between AA and HS, an exploratory receiver operating characteristic curve (ROC) analysis was conducted to compare the two groups. The analysis revealed that Galectin-10 exhibited the highest discriminatory performance for distinguishing AA from HS (Area under the curve, AUC = 0.95), followed by ECP (AUC = 0.82), and EDN (AUC = 0.72), as shown in Figure 4.

**Figure 5:**
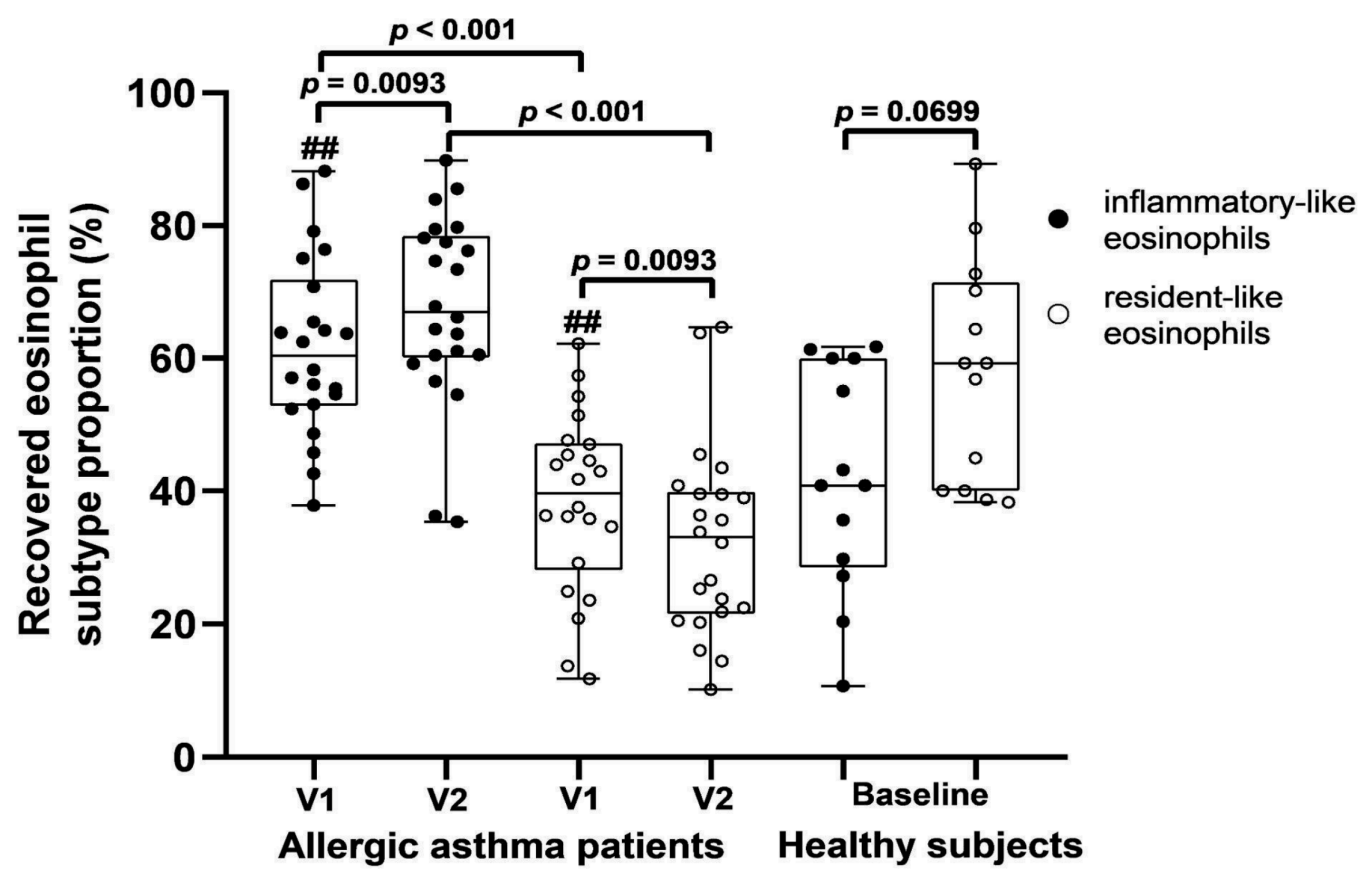
Recovered blood iEOS-like and rEOS-like cell proportions before and after bronchial allergen challenge. Allergic asthma *n* = 22. Healthy subject *n* = 13. ## *p* < 0.01—compared with the respective blood eosinophil subtype of the healthy subject group. iEOS—inflammatory eosinophil, rEOS—resident eosinophil, FeNO—fractional exhaled nitric oxide, V1—first experimental visit, V2—second experimental visit—24 h after bronchial allergen challenge.

Using magnetic separation, we isolated blood iEOS-like and rEOS-like cells and calculated their proportions relative to the total. At baseline, in the AA group, 61.68 ± 2.87% of eosinophils were iEOS-like cells, a proportion significantly higher than in the HS group (42.04 ± 4.69%, *p* < 0.01). Twenty-four hours after BAC in the AA group, the iEOS-like cell count increased significantly (61.68 ± 2.87% vs. 67.47 ± 3.05%, *p* < 0.01). As shown in Figure 5. These values reflect the relative distribution of magnetically recovered eosinophil subtype fractions intended for gene expression analysis rather than for direct flow cytometric evaluation of peripheral blood eosinophils. Nevertheless, we used the same standardized blood eosinophil subtype isolation protocol for all individuals studied, enabling valid comparisons across participant visits and study groups.

**Figure 6:**
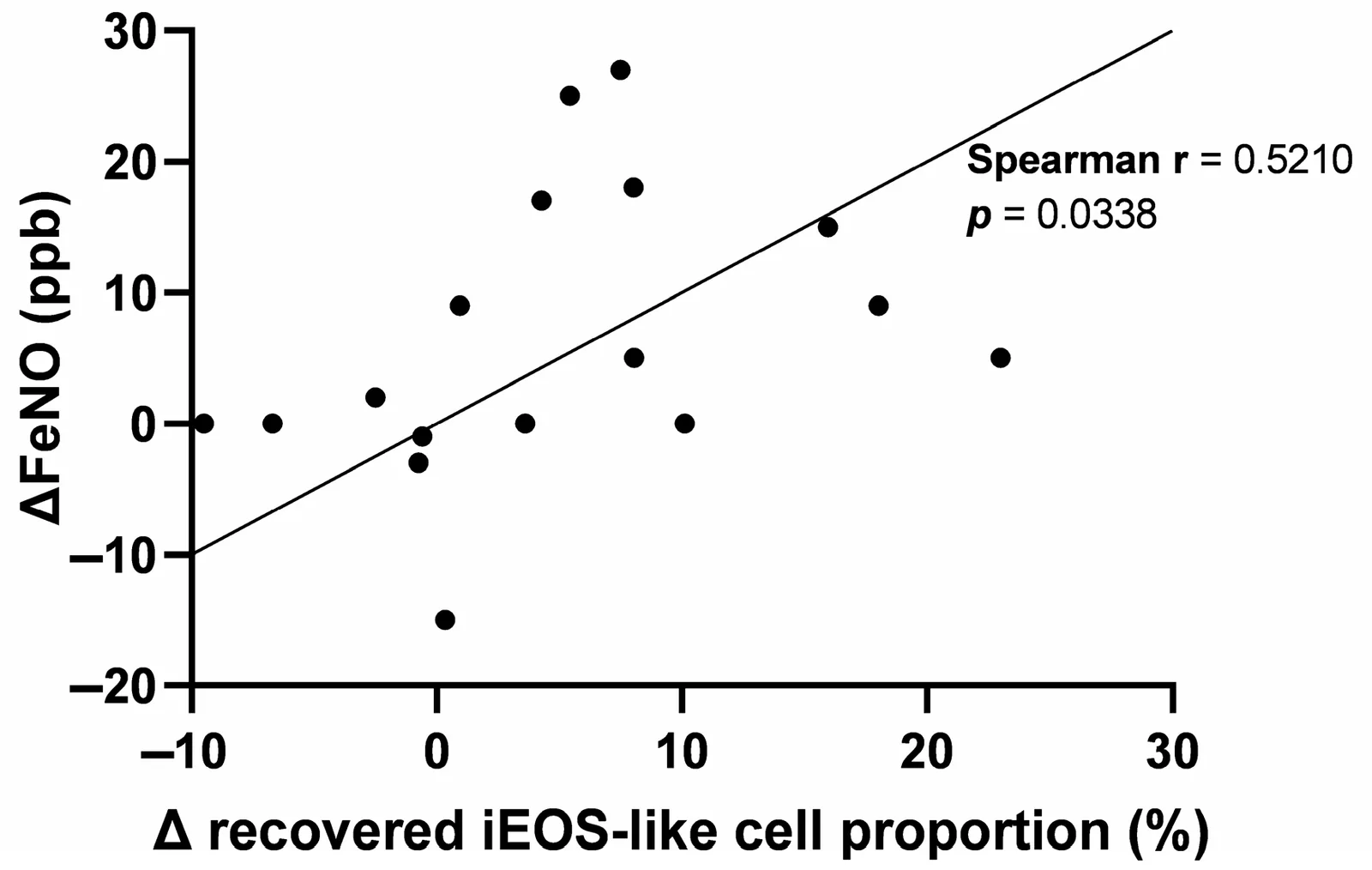
Spearman correlation between allergen-induced changes in recovered blood iEOS-like cell proportions and FeNO in allergic asthma patients. Allergic asthma patient *n* = 17. FeNO—fractional exhaled nitric oxide. iEOS—inflammatory eosinophil. Δ denotes calculation within the same study subject’s 24 h after BAC value (V2) subtracted from baseline before BAC (V1). The *X*-axis values are expressed as differences in percentage points, not relative percentage change.

In the AA group, baseline and post-BAC blood iEOS-like cell proportions showed no significant correlations with circulating concentrations of ECP, EDN, NOX2, or Gal10, lung function, AHR, or total IgE. The only moderate, positive, and significant correlation was between changes in FeNO and changes in recovered iEOS-like cells after BAC (Spearman r = 0.52, *p* = 0.034), as shown in Figure 6.

## 4. Discussion

This study found that BAC with *D. pteronyssinus* allergen shifts the blood eosinophil subtype distribution toward iEOS-like cells and promotes expression of granule protein genes, while serum ECP, EDN, and Gal10 concentrations increase in patients with AA. Allergen-induced changes in recovered iEOS-like cell proportions were associated with increased FeNO, but not with circulating mediators or other clinical parameters. Serum ECP, EDN, and Gal10 were higher at baseline in AA than in HS, and exploratory ROC analysis indicated that Gal10 provided the strongest discrimination between the two groups. Finally, blood eosinophil subtypes from asthmatic patients show increased expression of secondary granule proteins and enzymes involved in cysteinyl leukotriene production compared with eosinophils from healthy subjects, and iEOS-like cells display enhanced *CLC* and *EPX* expression in the AA group compared to rEOS-like cells.

Eosinophils contain various tissue-damaging cationic granule proteins stored in their specific granules. These proteins are known to contribute to host resistance against helminths, bacterial, viral, and fungal pathogens [35]; however, they may also affect other structural cells—airway epithelial cells, smooth muscle cells, fibroblasts, and extracellular matrix—at sites of inflammation, thus contributing to structural airway changes and airflow limitation [1,10,36]. Accordingly, eosinophil-derived granule proteins have been proposed as potentially useful biomarkers for diagnosing asthma [37], assessing disease severity [28,38,39], or monitoring anti-eosinophil treatment [40,41]. In this study, blood iEOS-like and rEOS-like cells from AA patients showed higher expression of *MPB*, *EDN* (*RNASE2)*, *ECP* (*RNASE3*), *EPX*, and *CLC* than those in the HS group, indicating that these cell types are transcriptionally active despite residing in peripheral blood rather than in tissues. Notably, AA group iEOS-like cells exhibit higher *CLC* and *EPX* expression than rEOS-like cells, supporting eosinophil subtype-specific functional specialization [17,42]. Consistent with the gene expression findings, the observed blood serum ECP, EDN, and Gal10 concentrations were significantly higher in the AA group than in the HS group, while an exploratory ROC analysis indicated that Gal10 had the highest discriminative performance between the groups analyzed, followed by ECP and EDN. Overall, the findings indicate that blood eosinophils may undergo molecular activation in AA, and that circulating eosinophil-derived proteins may provide complementary information for distinguishing asthma.

Asthma is a heterogeneous disease because its pathogenesis can be driven by various inflammatory or structural lung cell types and their secreted mediators [43]. Thus, experimental models that induce a controlled, reproducible allergic inflammatory response are essential for investigating changes in inflammatory immune cell activation and the release of their contents in vivo. BAC models provide a controlled experimental framework for investigating the mechanisms of acute and late-phase allergen-induced airway inflammation in humans. In this study, 24 h after BAC, the relative proportion of circulating blood iEOS-like cells increased, followed by enhanced expression of *CLC*, *MBP*, and *TGF-β1* in both subtypes, whereas *EPX* and *NOX2* increased only in iEOS-like cells. Additionally, serum EDN and ECP concentrations also increased despite no corresponding changes in EDN and ECP gene expression. Eosinophils can release granule contents through classical or compound exocytosis, piecemeal degranulation, and cytolysis [44], and the present serum measurements cannot identify which release pathway predominated after BAC. Interestingly, Gal10 showed a distinct response: unlike EDN and ECP, it is not stored within secondary granules but is concentrated in the eosinophil cytoplasm and is not considered to undergo piecemeal degranulation or compound exocytosis, as it is associated with eosinophil cytolysis, eosinophil extracellular trap, and CLC formation [45,46]. Meanwhile, preformed cationic proteins, such as EDN and ECP, are stored in eosinophil secondary granules; thus, the observed gene expression pattern is consistent with rapid mobilization of existing intracellular stores rather than an immediate requirement for new transcription. Similarly, the increase in MBP transcript expression after BAC is better interpreted as transcriptional upregulation rather than as direct evidence of protein release. Overall, these findings indicate that eosinophil-derived mediators are regulated by uncoupled mechanisms of transcriptional activation and release during acute allergen exposure.

Nitric oxide is an important regulator of immune responses and a marker of airway inflammation, which can be overproduced in asthma [47]. Although type 2 inflammation is prevalent in asthma, it can be complex and heterogeneous because no single biomarker fully captures both airway and circulating inflammatory activity [28,48,49]. In this study, the HDM allergen-induced changes in iEOS-like cell distribution were positively associated with changes in FeNO but not with the investigated eosinophil serum mediators, clinical lung function parameters, airway hyperresponsiveness, or total IgE. Recent studies note that changes in FeNO may indicate type 2 airway inflammation that is not reflected systemically in mild asthma [50]. In addition, a recent congress abstract examined early- and late-onset asthmatics and found no relationship between FeNO and iEOS (defined as Singlec-8^+^CD16^−^CD62^low^) [51]. In contrast, our paired BAC model in the mild-to-moderate AA group observed an association between changes in recovered iEOS-like cells and FeNO. This difference may indicate that a controlled longitudinal BAC model can capture within-individual responses that are not apparent in cross-sectional measurements. The finding merits cautious interpretation, suggesting that eosinophil subtype distribution may parallel the airway type 2 inflammatory response, whereas serum eosinophil-derived mediators reflect a distinct component of eosinophil granule content release. Nonetheless, given the exploratory nature of the analysis and the modest sample size, this relationship should not be taken as evidence that iEOS-like cells outperform total eosinophil counts as a validation biomarker. Larger studies with paired airway-derived samples and direct eosinophil subtype flow cytometry analysis using additional surface markers [17] are warranted.

Partially distinct transcriptional and serum responses may indicate that blood eosinophil gene expression and the investigated mediator concentrations capture different stages and compartments of eosinophil activation. Previous studies have linked eosinophilic airway inflammation to local oxidative stress, including associations with exhaled hydrogen peroxide, sputum eosinophilia, and eosinophil-related oxidative responses during virus-induced asthma exacerbations [52,53]. However, circulating NOX2 protein remains poorly characterized in asthma; we investigated it in this study. We found that NOX2 transcript expression was higher in eosinophil subtypes from AA patients than in those from the HS group, and that its expression further increased after BAC only in iEOS-like cells, a pattern compatible with upregulation of oxidative pathways during the late-phase allergic response [3,54]. Nevertheless, this study did not measure cellular ROS production or NOX2 enzymatic activity, and the lack of a corresponding increase in serum NOX2 further suggests that circulating immunoreactive NOX2 does not directly reflect eosinophil NOX2 transcription or intracellular NADPH oxidase activity [55]. A similar distinction between eosinophil transcriptional activation and downstream functional mediator production may apply to the 5-lipoxygenase pathway. The observed increase in *ALOX5* expression in blood iEOS-like and rEOS-like cells from patients with AA compared with the HS group, with unchanged *LTA4H* and *LTC4S* expression, suggests selective transcriptional priming of the 5-lipoxygenase pathway rather than coordinated activation of leukotriene synthesis. Consistent with this, Sunata et al. reported increased *ALOX5* expression in human blood eosinophils from patients with asthma-chronic obstructive pulmonary disease (COPD) overlap and eosinophilic COPD, without corresponding significant increases in most measured 5-lipoxygenase-derived lipid mediators [56]. Since the present study did not evaluate enzyme activity or leukotriene concentrations, it cannot infer increased functional leukotriene production. Therefore, upregulation of *ALOX5* expression may reflect pathway-level transcriptional changes in eosinophils from the AA group, but its functional significance warrants careful interpretation.

A potential limitation of this study relates to the blood eosinophil subtype isolation strategy. After total eosinophil purification, we isolated rEOS-like (CD62L^+^) cells by positive selection and labeled the unselected cells as iEOS-like (CD62L^−^). Recent similar studies further distinguish iEOS as CD62L^low^ and rEOS as CD62L^bright^ cell populations, rather than strictly CD62L-negative and CD62L-positive subsets [57]. Therefore, some overlap between isolated populations cannot be ruled out, and the reported proportions in this study should be interpreted as relative distributions of recovered cell-subtype fractions rather than as absolute in vivo blood values. Moreover, magnetic-activated cell sorting relies on sufficient CD62L surface expression; eosinophils with low CD62L expression were likely recovered with the CD62L^−^ cell population. Nevertheless, the observed increase in the proportion of blood iEOS-like cells in AA is consistent with previous studies employing flow cytometric CD62L-based phenotyping [57,58].

Another limitation is that this study evaluated BAC response at a single 24 h time point. As a result, the study could not assess early dynamic changes and relationships among eosinophil transcriptional activation, degranulation, and circulating protein concentrations during the immediate and intermediate phases of inflammation. Nonetheless, we selected the 24 h time point to investigate established late-phase allergen-induced inflammation, characterized by eosinophil activation, recruitment, and accumulation within the inflamed airway [59]. Moreover, the relatively small sample size may limit the generalizability of between-group comparisons. Although median age, sex distribution, and BMI were broadly comparable between AA and HS, the asthma group had wider age and BMI ranges, including four participants with obesity. The analyses were not adjusted for age, sex, or BMI; thus, residual effects of these factors on blood iEOS-like and rEOS-like cell function cannot be excluded. All individuals in the AA group had a non-severe disease course and were sensitized to at least *D. pteronyssinus*, and the group showed elevated FeNO, blood eosinophil counts, and serum IgE, consistent with a biomarker profile of prominent type 2 inflammation. In addition, we did not formally classify individuals into T2-high, T2-low, or mixed inflammatory endotypes; thus, some inflammatory heterogeneity may remain. Still, strict, predefined inclusion and exclusion criteria partially limited heterogeneity, and the controlled longitudinal BAC model enabled paired within-participant assessment, reducing the influence of stable interpersonal factors.

Finally, gene expression analysis of blood eosinophil subtypes was not complemented by direct protein assessment in the same isolated cell populations. Transcriptional changes do not necessarily reflect intracellular protein levels or mediator release, as post-transcriptional and post-translational regulatory mechanisms can substantially influence protein expression [60,61]. Gene expression profiling and circulating eosinophil-derived serum mediators were evaluated as complementary molecular and functional aspects of eosinophil biology, providing a rationale for future analyses of eosinophil-derived proteins.

## 5. Conclusions

We demonstrate that BAC induces a late-phase eosinophilic response in AA characterized by a partially eosinophil subtype-specific increase in gene expression and in circulating eosinophil-derived mediators. The increase in Gal10 across both *CLC* transcript levels in eosinophils and in serum concentration, but not in ECP or EDN, suggests mediator-specific mechanisms that regulate gene expression and extracellular release. Blood eosinophil subtypes in AA show increased granule protein gene expression over HS, particularly in iEOS-like cells, consistent with cell-subtype functional activity. The association between the in vivo BAC-induced increase in the proportion of recovered iEOS-like cells and changes in FeNO was positive, but not with changes in serum ECP, EDN, or Gal10. This suggests that cellular and soluble biomarkers may capture complementary aspects of allergen-induced eosinophilic inflammation and support further investigation in larger, phenotypically distinct asthma cohorts and paired airway-derived samples.

## Figures and Tables

**Table 1:** Inclusion and exclusion criteria for the study.

Inclusion Criteria		Exclusion Criteria (Both Groups)
Allergic Asthma Patients	Healthy Control Subjects	
− Asthma diagnosis − Non-severe course of the disease − Positive skin prick test to at least *Dermatophagoides pteronyssinus* allergen − Positive methacholine challenge or bronchial reversibility test	− No chronic respiratory and other diseases − Negative skin prick test	− Age < 18 years − Clinically significant allergy symptoms − Active airway infection ≤ 1 month prior to study − Asthma exacerbation ≤ 1 month prior to study − Use of oral steroids ≤ 1 month prior to study − Active smoking (use of any inhaled nicotine-containing products: vaping devices, cigarettes, electronic cigarettes, heated tobacco products) − Former smoking (≥100 lifetime combustible cigarettes) or previous regular use of electronic cigarettes, vaping devices, heated tobacco products

**Table 2:** Demographic and clinical characteristics of Study Subjects.

	AA Patients		Healthy Subjects
Number, *n*	23		13
Sex, male/female, *n*	9/14		5/8
Age, median (range), years	25.5 (18–66)		24 (22–34)
BMI, median (range), kg/m^2^	23.3 (17.4–40.6)		22.10 (17–29.6)
Sensitization to *D. pteronyssinus*, *n*	23		NR
PD20_m_, mg	0.372 ± 0.065		NR
PD20_a_, HEP/mL	11.06 ± 2.01		ND
	V1, 0 h baseline	V2, 24 h after BAC	
FEV_1_, L	3.22 ± 0.16 #	3.04 ± 0.17 **	4.15 ± 0.26
FEV_1_, % of predicted	88.55 ± 2.78 ###	81.52 ± 3.31 ****	105.1 ± 2.82
FVC, L	4.18 ± 0.20	4.01 ± 0.19 **	4.75 ± 0.33
FVC, % of predicted	95.29 ± 2.01	86.66 ± 4.53 ***	100.2 ± 2.57
FEV_1_/FVC, %	77.70 ± 1.73 ##	74.05 ± 2.02 **	87.21 ± 2.18
Blood eosinophil count, (×10^9^)/L	0.378 ± 0.046 ####	0.451 ± 0.053 *	0.105 ± 0.010
Blood eosinophil count, %	6.20 ± 0.75 ####	7.49 ± 1.07 *	1.79 ± 0.30
IgE, IU/mL	288.0 ± 67.6 ####	274.8 ± 62.2	13.2 ± 3.9
FeNO, ppb	73.7 ± 14.8 ####	83.9 ± 15.8 **	11.7 ± 1.9

AA—allergic asthma. BAC—bronchial allergen challenge. BMI—body mass index. *D. Pteronyssinus*—*Dermatophagoides pteronyssinus*. FeNO—fractional exhaled nitric oxide. FEV1—forced expiratory volume in one second. IgE—immunoglobulin E. ND—not done. NR—no response. PD20_a_—provocation dose of *D. pteronyssinus* allergen resulting in a 20% decrease in FEV_1_. PD20_m_—provocation dose of methacholine resulting in a 20% decrease in FEV1. Data presented as mean ± SEM. # *p* < 0.05, ## *p* < 0.01, ### *p* < 0.001, #### *p* < 0.0001, compared with the HS group; * *p* < 0.05, ** *p* < 0.01, *** *p* < 0.001, **** *p* < 0.0001, compared within the AA group before BAC.

## Data Availability

The original contributions presented in this study are included in the article. Further inquiries can be directed to the corresponding author.

## Abbreviations

The following abbreviations are used in this manuscript:

AA	Allergic asthma
AHR	Airway hyper-responsiveness
ALOX-5	Arachidonate 5-lipoxygenase
AUC	Area under the curve
BAC	Bronchial allergen challenge
CBC	Complete blood count
CLC	Charcot-Leyden crystal
COPD	Chronic obstructive pulmonary disease
ECP	Eosinophil cationic protein
EDN	Eosinophil-derived neurotoxin
ELISA	Enzyme-linked immunosorbent assay
EPX	Eosinophil peroxidase
FeNO	Fractional exhaled nitric oxide
FEV_1_	Forced expiratory volume in 1 s
FVC	Forced vital capacity
Gal10	Galectin-10
HDM	House dust mite
HEP	Histamine-equivalent prick
HS	Healthy subject
iEOS	Inflammatory eosinophil
IgE	Immunoglobulin E
IL	Interleukin
ILC	Innate lymphoid cell
LTA4H	Leukotriene A4 hydrolase
LTC4S	Leukotriene C4 synthase
LUHS	Lithuanian University of Health Sciences
MBP	Major basic protein
mRNA	Messenger ribonucleic acid
NOX2	Nicotinamide adenine dinucleotide phosphate oxidase 2
OD	Optical density
PD20_m_	Provocation dose of methacholine causing a 20% decrease in FEV_1_
qRT-PCR	Quantitative reverse transcriptase polymerase chain reaction
rEOS	Resident eosinophil
ROC	Receiver operating characteristic curve
SEM	Standard error of the mean
TGF	Transforming growth factor
Th2	T helper type 2 cells
V1	First experimental visit
V2	Second experimental visit—24 h after bronchial allergen challenge

## Appendix A. Serum Mediator Analysis

**Table A1:** Serum Mediator Concentration Analysis.

	Allergic Asthma V1	Allergic Asthma V2	Healthy Subjects	Allergic Asthma V1 vs. V2	Allergic Asthma V1 vs. Healthy Subjects	Allergic Asthma V2 vs. Healthy Subjects
Serum EDN, ng/mL	28.72 ± 1.78	32.14 ± 2.46	23.06 ± 1.46	*p* = 0.0174	*p* = 0.0389	*p* = 0.0052
Serum Galectin-10, ng/mL	5.71 ± 0.45	5.85 ± 0.46	3.06 ± 0.21	*p* = 0.0337	*p* < 0.0001	*p* < 0.0001
Serum NOX2, ng/mL	4.38 ± 0.77	3.89 ± 0.67	5.07 ± 0.77	*p* = 0.9170	*p* = 0.4083	*p* = 0.2629
Serum ECP, ng/mL	4.96 ± 0.22	5.79 ± 0.44	4.21 ± 0.11	*p* = 0.0198	*p* = 0.0023	*p* < 0.0001

Results are shown as mean ± SEM. Allergic asthma *n* = 17; Healthy subjects *n* = 13. EDN—eosinophil-derived neurotoxin, NOX2—nicotinamide adenine dinucleotide phosphate oxidase 2, ECP—eosinophil cationic protein. V1—allergic asthma patient’s first experimental visit. V2—allergic asthma patient’s 2nd experimental visit, 24 h after a bronchial allergen challenge with house dust mite. Differences between allergic asthma and healthy subject groups were evaluated using the two-sided Mann–Whitney U test. The Wilcoxon matched-pairs signed-rank test was used to compare differences within the allergic asthma group.
